# MicroRNAs in Axial Spondylarthritis: an Overview of the Recent Progresses in the Field with a Focus on Ankylosing Spondylitis and Psoriatic Arthritis

**DOI:** 10.1007/s11926-021-01027-5

**Published:** 2021-07-03

**Authors:** Francesca Motta, Andrea Pederzani, Maria Cristina Carena, Angela Ceribelli, Paul B. Wordsworth, Maria De Santis, Carlo Selmi, Matteo Vecellio

**Affiliations:** 1 Division of Rheumatology and Clinical Immunology, IRCCS Humanitas Research Hospital, Rozzano, Milan, Italy; 2 Department of Biomedical Sciences, Humanitas University, Pieve Emanuele, Milan, Italy; 3 Leukocyte Biology Unit, IRCCS Humanitas Research Hospital, Rozzano, Milan, Italy; 4 Nuffield Department of Orthopaedics, Rheumatology and Musculoskeletal Sciences, University of Oxford, Oxford, UK

**Keywords:** MicroRNA, Spondylarthritis, Psoriatic arthritis, Biomarkers

## Abstract

**Purpose of Review:**

To highlight the recent discoveries and lines of evidence on the role of microRNAs in ankylosing spondylitis (AS) and psoriatic arthritis (PsA), focusing on their expression profiling and mechanisms of action.

**Recent Findings:**

AS and PsA are chronic inflammatory musculoskeletal diseases with axial manifestations and represent an excellent model for studying microRNAs contribution to the disease pathogenesis, particularly through immunomodulation, inflammation, and bone remodelling, or their value as candidate diagnostic and prognostic biomarkers.

**Summary:**

MicroRNAs are single-stranded nucleotides able to regulate gene expression. They are a key component of the epigenetic machinery, involved in physiological and pathological processes. The contribution of microRNAs in AS and PsA (such as miR-29a in regulating bone metabolism) is highlighted by several works in the field but their utility as possible markers must be still confirmed, particularly in larger patients’ cohorts.

## Introduction

Spondyloarthritis (SpA) is a heterogenous group of rheumatic diseases encompassing ankylosing spondylitis (AS), psoriatic arthritis (PsA), reactive arthritis, arthritis associated with inflammatory bowel disease (IBD), a subgroup of juvenile idiopathic arthritis, and undifferentiated spondyloarthritis [1–3]. These disorders share clinical and genetic features such as enthesitis, dactylitis, uveitis, inflammation of the spine (specifically sacroiliac joints [SIJ] and spine), and association with the HLA-B27 allele [4, 5]. Among these disorders, AS and PsA are the most paradigmatic, predominantly affecting the peripheral joints and the axial skeleton, respectively, although with significant clinical overlapping [6, 7].

AS typically develops in males in their third decade of life and affects mainly the SIJ and the spine symmetrically [8, 9]. It usually presents with inflammatory low back pain, progressive stiffness, and impaired range of motion of the spine, which are the manifestation of inflammation of the axial skeleton, erosive destruction of cartilage and bone, progressive new bone formation at entheseal sites, and syndesmophyte development, ultimately resulting in the typical “bamboo spine” [10, 11]. While in some patients structural damage can be detected by X-ray scans after a few years from the onset of the disease, the assessment of inflammation is better performed with magnetic resonance (MRI). In fact, especially at early stages, MRI can detect inflammatory alterations years before radiographic changes become apparent [9, 12]. The term axial SpA (axSpA) refers to both forms of the disease: the radiographic form (AS) and the non-radiographic one (nr-axSpA). Both are now considered together in the current management recommendations, which include a non-pharmacological and a pharmacological approach [13]. Among approved therapies, the introduction of biological disease-modifying antirheumatic drugs (bDMARDs) targeting TNF-α and IL-17 has dramatically changed the prognosis since synthetic DMARDs are ineffective [13, 14]. As a consequence, there is a strong need to rely on specific biomarkers to support the diagnosis, to monitor the disease, and to predict the response to treatment [15].

PsA develops in up to 30% of patients with psoriasis, being more frequent in those with nail lesions. It may affect both the peripheral joints and the axial skeleton, but also entheses are frequently involved [16, 17]. Axial manifestations occur in 5–28% of patients with early onset and in 25–70% of patients with longstanding disease [18], differing from AS for the asymmetric involvement of SIJ [19], the distribution of syndesmophytes, which typically progress randomly along the spine and have a “chunky” morphology, and the weaker association with HLA-B27 [17]. The diagnosis of PsA relies on classification criteria which include clinical, laboratory, and imaging features [20], as specific biomarkers are lacking. MRI allows early recognition of inflammatory axial changes [21, 22]. Similar to AS, early management is recommended according to disease phenotype and in axial involvement bDMARDs substantially modify the outcome [23] and the quality of life.

## Currently Available Biomarkers in AS and PsA

A range of different potential biomarkers have been studied over the recent years in AS and PsA, including genetic, transcriptomic, proteomic, metabolomic, and microbiome biomarkers [15].

The biomarkers for AS and PsA currently used in the daily routine medical practice are the genetic test for HLA-B27 and non-specific acute phase reactants as C-reactive protein (CRP) and erythrosedimentation rate (ESR).

HLA-B27 has a prominent role in the diagnosis and classification of low back pain [24] and is the only known genetic marker common to AS and PsA with axial involvement, as it is present in more than 80% of patients with AS [25], ranges from 20 to 35% in PsA, and reaches a prevalence of approximately 50% in cases of axial PsA [26]. It also provides prognostic information on disease phenotype, spine involvement, and radiographic damage [27]. Nonetheless, it is not useful to predict disease activity or the response to specific treatments.

To assess the activity, the Ankylosing Spondylitis Disease Activity Score (ASDAS) [28] for AS and the Disease Activity in Psoriatic Arthritis (DAPSA) for PsA [29] take into account clinical features and CRP or ESR and are validated tools to monitor disease activity, despite showing some limitations [30]. Imaging is an essential resource in the diagnosis, as the detection of radiographic sacroiliitis or of active inflammation of SIJ at MRI represents a criterion for classification of axSpA [24]. Moreover, it allows to monitor inflammation and structural damage [31]. Nonetheless, earlier and finer recognition of changes in disease course is needed, preferably with markers that reflect MRI inflammation in the spine [32].

For these reasons, one of the most important unmet needs in AS is the identification of reliable markers or the improvement of existing ones, for use in research and in clinical practice, able to help for an early diagnosis, to reflect axial inflammation for monitoring the disease, to provide prognostic information, and to predict the response to different therapies [32].

## Genetics of AS and PsA

AS has a high degree of heritability, estimated between 32 and 69% [33]. AS is frequently associated with HLA-B27, which explains about 20% of its heritability, while other loci account for only 4.3% [34]. About 140 subtypes of HLA-B27 have been identified, among which B*2702, B*2703, B*2704, B*2705, and B*2710 are reported to increase the risk of developing AS [35]. Other HLA loci have also been implied [33].

Thanks to genome-wide association studies and Immunochip data, important progress has been made in the last decade with the discovery of genetic association with AS beyond HLA. To now, these studies led to the identification of 113 loci associated with the disease [36, 37].

Genome-wide association studies have shown the implication of several genetic loci, some of them having pleiotropic effects. In 2013, the study from the International Genetics of Ankylosing Spondylitis (IGAS) Consortium demonstrated that several AS-related gene loci are involved in different immunomodulatory pathways, evaluating 10,619 patients with AS and 15,145 controls. Among the genes identified, several were related to the IL-17/IL-23 axis encoding for cytokines and cytokine receptors (*IL23R*, *IL12B*, *IL1R2*, *IL27*). Th17 effector cells develop from naïve T cells when the expression of the IL-23 receptor (IL-23R) is induced by both TGF-β and pro-inflammatory cytokines such as IL-6 and IL-1β [10, 38]. The identification of other genetic associations downstream of the IL-23R and within the Th17 lymphocyte functional and developmental pathway, such as *TYK2* (Tyrosine Kinase 2), *IL6R*, and *IL1R1*, confirmed the implication of the IL-23-driven pathway in AS [10, 12, 38]. Other genetic associations were reported in proximity of the nuclear factor kappa B (*NF-kB*), genes involved in cytokines’ production (*PTGER4*, prostaglandin receptor EP4 and *TNFRS1A*, TNF Receptor Superfamily Member 1A) and protein degradation (*UBE2E3*, Ubiquitin-conjugating enzyme E2 E3). Further, other genes shape T cell development and activity, such as CD8+ T cells (*IL7R*, *RUNX3* Runt-related transcription factor 3, *EOMES*, eomesodermin) [10, 34].

Last, one of the strongest and most studied associations (p=10^-50^) with AS, falling outside the MHC region, is with *ERAP1* (endoplasmic reticulum aminopeptidase 1) which is involved in processing peptide antigens for presentation by MHC class 1 molecules. Together with *ERAP1*, also *ERAP2* and *NPEPPS* (puromycin-sensitive aminopeptidase) are strongly associated with AS [25, 33]. These are involved in trimming peptides before presentation by MHC class 1, and in the processing of proteasome-derived peptides, respectively [25, 33].

In PsA, the genetic component is supported by the observation that first-degree relatives of patients have a relative risk of 39 to develop PsA, which decreases to 2.3 in fourth-degree relatives [39]. Similar to AS, PsA is associated with HLA-B27, with a prevalence of 20–50% [26, 40] and this allele, together with HLA-B08, HLA-B38, and HLA-B39, is linked to the axial manifestations of the disease [40, 41]. HLA-B27 has also been linked with a form of early-onset PsA with severe peripheral damage [25, 40].

Recent genome-wide association studies highlighted the contribution of several non-MHC genes in development of the disease, such as *TRAF3IP2*, *TNIP1*, *REL*, *IFNLR1*, *IFIH1*, and *NFKBIA* which are also implicated in psoriasis [42]. Polymorphisms in killer cell immunoglobulin-like receptor (KIR) genes, such as *KIR2DS2*, were present in patients with PsA but not in patients with only cutaneous manifestations [43]. KIRs are present on natural killer cells when they interact with MHC class 1 molecules [25]. An IL-13 polymorphism was also found in patients with PsA but not with psoriasis [40].

PsA GWAS suggested that there are PsA-specific genetic variants independent of those previously identified in isolated psoriasis, near *IL23R* and *TNFAIP3* (TNFα-Induced Protein 3) genes. Further, the association with *ERAP1* and *ERAP2*, *TLR4*, and *RUNX3*, also found associated with AS, highlights the common grounds between these two disorders [44].

## MicroRNAs as Biomarkers in axSpA and PsA

MicroRNAs are a group of endogenous non-coding RNAs, on average spanning 22 nucleotides, acting as post-transcriptional regulators of gene expression by targeting messenger RNAs (mRNAs) and inducing their cleavage, repressing their translation, or promoting mRNA decay [45, 46]. MicroRNAs were first observed in 1993, controlling the timing of larval development in *Caenorhabditis elegans* [47], and play an important role in human diseases, including cancer and heart diseases, whereas recent studies have highlighted their contribution to rheumatic diseases [48]. In the present review, we have highlighted the most recent discoveries in the field of microRNAs in axSpA and PsA, reporting plausible evidences for the development of future biomarkers for axial disease [49, 50].

### MicroRNAs in AS (Table 1)

The first study on the microRNA expression profile in T cells in AS and healthy controls was performed in 2003 by Lai and his group. The authors demonstrated that the expression of three microRNAs, miR-16, miR221, and let-7i, was higher in T cells of patients with AS compared to controls, and correlated with the Bath Ankylosing Spondylitis Radiology Index of the lumbar spine [51]. More recently, a study by Li et al. reported an elevated expression of miR-17-5p, miR-27a, miR-29a, and miR-126-3p expression in peripheral blood mononuclear cells (PBMCs) of patients with AS compared to healthy control [53]. The presence of mir-29 in AS peripheral blood has been highlighted by different studies, particularly evidencing the role of miR-29a in regulating bone metabolism. miR-29a enables negative regulation of Dkk-1 expression and facilitates activation of the Wnt pathway, resulting in increased bone formation [53, 54].

**Table 1:** MicroRNAs investigated in AS

MicroRNA	Target	MicroRNA expression	Sample	Effect	References
miR-16	Bcl-2	Increased	T cells	-	[51]
miR-221	c-kit	Increased	T cells	-	[51]
let-7i	TLR-4,	Increased	T cells	Decreased expression of IFN-γ,	[51]
	IGF1R			Decrease in phosphorylation of mTOR and Akt, inducing autophagy	[52]
miR-29a	DKK1	Increased	PBMCs, hMSCs	Facilitate activation of the WNT pathway	[53–55]
miR-130a	TNF-1α	Decreased	PBMCs	Increased expression of TFN-1α	[56]
miR-146a, miR-155	-	Increased	Serum	Increased CRP levels (miR-155)	[57]
miR-214	PTEN	Increased	Synovial fluid, blood	Promote osteoclast activity	[58]
miR-4284	CXCL5	Decreased	hMSCs	Inhibit osteoclastogenesis	[59]
miR-124	ANTXR2	Increased	Blood	Promote JNK activation, inducing autophagy	[60]

The expression of mir-29a was found upregulated in PBMCs of patients with AS when compared with patients with rheumatoid arthritis and healthy controls, thus suggesting a plausible implication of miR-29a as a diagnostic marker for new bone formation in AS [55]. A clinical trial involving miR-29 is currently ongoing. Remlarsen, also called MRG-201, is a phase 2 study designed to mimic the activity of miR-29 in collagen and connective tissue diseases, with the aim to evaluate the expression of collagen and other proteins involved in scar formation. The outcomes may be important for scleroderma patients [61].

A work published in 2018 investigated the microRNA expression in CD14+ monocytes and CD4+ T lymphocytes isolated from 81 axSpA patients and 55 controls. The authors identified thirteen microRNAs consistently deregulated in CD14^+^ cells with miR-361-3p, miR-223-3p, miR-484, and miR-16-5p being the most differentially expressed. In CD4^+^ T cells, 11 microRNAs were differentially expressed between patients and controls, with miR-16-1-3p, miR-28-5p, mir-146a-5p, miR-199a-5p, and miR-126-3p being the most strongly upregulated in axSpA patients. In particular, mir-146a-5p levels seem inversely correlated with CRP levels and ASDAS in patients with axSpA [62].

An important study on the role of long non-coding RNAs (lncRNA) MEG3 was recently published. lncRNAs are RNAs longer than 200 nucleotides, not translated into functional proteins. The large family includes lncRNAs transcribed by RNA polymerase II and other RNA polymerases, lncRNAs from intergenic regions (called lincRNAs), and sense or antisense transcripts overlapping with other genes [63]. The authors discovered that lncRNA MEG3 expression was downregulated in patients with AS and that MEG3 was negatively correlated with the level of inflammatory cytokines, such as of IL-1β, IL-6, and TNF-α. The authors found that the lncRNA may interact with miR-146a, which is highly expressed in patients with AS’ serum and positively correlated with inflammatory cytokine level. The authors suggested MEG3 may inhibit the expression of miR146-a, making it a possible attractable target for AS therapy [64].

Epigenetic regulatory enzymes were also investigated as modulators of microRNAs. A study by Jiang and colleagues observed increased histone deacetylase-3 (HDAC3) and decreased miR-130a expression levels in PBMCs from patients with AS. Chromatin immunoprecipitation assay revealed that, in patients with AS, HDAC3 can be recruited to the promoter region of the gene encoding miR-130a. HDAC3 inhibition or knockdown resulted in increasing the expression of miR-130a and conversely in decreasing TNF-1α expression (both mRNA and protein) in PBMCs. These findings suggested HDAC3 having a central role in regulating miR-130a expression via its target TNF-1α: these mechanisms might play a role at the interface between epigenetics and inflammation in the AS pathophysiological process [56].

The potential of microRNAs as future biomarkers for diagnosis of AS seems promising. In a recent study, Yang and colleagues showed that the expression of a microRNA signature (in particular miR-335–5p, miR-27a, and miR-218) together with the presence of smoking history, HLA-B27 positivity, elevated ESR, serum CRP concentration, and “average sacroiliitis” appeared to represent a good model to assess the presence of syndesmophytes in patients with AS (AUC 0.97) [65]. An RNAseq approach followed by qPCR showed the upregulation of miR-146a and miR-155 in serum of patients with AS, compared to healthy controls [57]. Further, the concentration of miR-155 was found correlated with CRP concentrations and BASDAI (Bath Ankylosing Spondylitis Disease Activity Index) and mSASSS (modified Stoke Ankylosing Spondylitis Spinal Score) values, suggesting a plausible utility for miR-155 as a marker of disease activity. A recent study performed on 800 patients with AS and healthy individuals provided important results for miR-146a-5p and miR-125a-5p, miR-151a-3p, miR-22-3p, miR-150-5p, and miR-451a86 as potential biomarkers of disease activity and structural damage [66]. The association of microRNAs with radiographic bone formation in axSpA was highlighted by a recent study showing the involvement of miR-29a-3p, miR-146a-5p, and miR-222-3p, all involved in extracellular matrix formation and spinal changes [67]. The involvement of microRNAs in bone regulation has been further highlighted in several works. Liu et al. found that the expression of RANKL and miR-214 in osteoblasts was increased upon stimulation by IL-17A: IL-17A-stimulated osteoblasts promoted the expression of miR-214 in osteoclasts, regulating their activity. The authors showed increased levels of IL-17A and miR-214 in the serum of patients with AS, compared to healthy controls. Further, the mRNA and protein expression levels of miR-214 were positively correlated with IL-17A levels both in blood and synovial fluid of patients with AS. These results suggest an inhibitory role in bone formation for miR-214 and recommend its potential diagnostic value [58].

The same authors reported osteoclastogenesis was inhibited in AS mesenchymal stem cells (MSCs) compared with health donors MSCs. This was largely caused by CXCL5, which was secreted significantly more in MSCs from patients with AS than healthy controls. Further, miR-4284 was found to interact with CXCL5, regulating its expression, confirmed also with experiments inhibiting miR-4284 [59].

Autophagy is a cellular mechanism consisting in delivering cytoplasmic organelles and macromolecules to lysosomes for destruction [68]. It is important in several cellular steps, including cell survival during short-term starvation, recycling of damaged organelles, and intracellular microbes’ clearance. In AS, plausibly autophagy process is associated with the removal of excess HLA class I heavy chains [69]. Hou and colleagues have shown that insulin-like growth factor 1 receptor (IGF1R) is a direct target of let-7i. When let-7i is overexpressed in T cells from patients with AS, IGF1R expression is markedly suppressed. The inhibition of IGF1R decreases of mTOR and Akt phosphorylation, with consequent downregulation of Bcl-2, upregulation of Bax, and cleavage of caspase 3 and PARP, leading to autophagy mechanisms, acting as a mechanism to protect T cells from apoptosis [52]. Further, Xia and co-authors found that miR-124 was upregulated in peripheral blood from patients with AS. The authors identified anthrax toxin receptor 2 (ANTXR2), a gene involved in new bone formation and strongly associated with AS [70], as target of miR-124. The authors also observed that the overexpression of miR-124 inhibited ANTXR2 expression. ANTXR2 inhibition by miR-124 promoted JNK activation in Jurkat cells, thus inducing autophagy [60].

### MicroRNAs in PsA (Table 2)

The role played by microRNAs in the pathogenesis of PsA, compared to the one in other autoimmune diseases such AS and rheumatoid arthritis, is less clear. The first study focusing on the microRNA expression profile in a cohort of early active patients with PsA was conducted by Ciancio et al. in 2017. The microRNA microarrays revealed a total of 19 deregulated microRNAs in PsA compared to healthy controls. In particular, the authors found miR-21-5p significantly upregulated in patients with PsA. Further, the same miR-21-5p was downregulated after 12 weeks of therapy, which correlated with reduction of DAPSA score [71]. A similar modification of miR-21 was observed in systemic sclerosis, another immune-mediated rheumatic disease. It was upregulated in diffuse disease, having a role in tuning collagen production by dermal fibroblasts, and its blockade was proposed as a therapeutic target [74].

**Table 2:** MicroRNAs in PsA

MicroRNA	Target	MicroRNA expression	Sample	Effect	References
miR-21-5p	-	Increased	PBMCs	-	[71]
miR-126-3p	*AKT2*, *PPP3CB*, *RANKL*, and *SDC2*	Decreased	PBMCs	Upregulation of SPP1	[72]
miR-23a	PDE4B	Decreased	Fibroblasts	Increased fibroblast migration and cytokine secretion	[73]

Pelosi and colleagues identified specific microRNA signatures associated with active PsA by performing microRNA microarrays in blood cells. The microRNAs identified are known to target important pathways involved in the pathogenesis of PsA, such as TNF (miR-130a-3p, miR-192-5p, and miR-199a-5), MAPK (miR-130a, miR-148a-3p, miR-192a-5p, miR-199a-3p, and miR-126-3p), and WNT (miR-130a-3p, miR-192-3p, and miR-192-5p) cascades. In particular, miR-126-3p was the most downregulated microRNA in patients with active disease, while its overexpression induced a decrease in mRNA transcript levels of several genes implicated in PsA, including AKT2, PPP3CB, RANKL, and SDC2 [72].

A recent study found that the synovial tissue expression of miR-23a was lower in PsA compared to osteoarthritis and was inversely correlated with disease activity and synovial inflammation. Further, TLR activation via polyinosinic:polycytidylic acid and lipopolysaccharide significantly decreased miR-23a expression, which led to increased synovial fibroblast migration and secretion of IL-6, IL-8, MCP-1, RANTES, and VEGF. The authors identified phosphodiesterase 4B (PDE4B) as the direct target of this microRNA and demonstrated that miR-23a-induced migration and enhanced cytokine expression were suppressed by blocking PDE4 signalling [73].

The first study to assess the ability of baseline microRNA expression to stratify patient outcomes to treatment was conducted by Wade and colleagues in 2020. Six microRNAs (miR-221-3p, miR-130a-3p, miR-146a-5p, miR-151-5p, miR-26a-5p, and miR-21-5p) were identified significantly higher in PsA compared to healthy controls. By analyzing responders versus non-responders, the authors demonstrated that higher baseline levels of miR-221-3p, miR-130a-3p, miR-146a-5p, miR-151-5p, and miR-26a-5p were associated with therapeutic response, according to the EULAR response criteria [75].

## Conclusions and Perspectives

Substantial efforts have been made to discover new biomarkers for the diagnosis, prognosis, and therapeutic response for AS and PsA in routine clinical practice. Analyzing the works proposed in this review, a significant weakness in study design emerged resulting in a very low rate of translation into clinical practice. In fact, there is an obvious real clinical utility in finding a specific microRNA or a microRNA signature as plausible diagnostic or prognostic biomarkers as comparing the profile of circulating microRNA levels between patients and controls might be promising at a first glance but limited prospective data have been obtained about microRNA profiles associated with specific phenotypes especially regarding disease severity.

Further independent and targeted studies are essential in the process of biomarker validation. The study design is crucial for future clinical utility of biomarkers. In particular, clearly defining the role of biomarkers (diagnostic or prognostic) must be followed by a reliable biomarker detection assay (i.e., RT-qPCR or immunohistochemistry assays for the identification of biomarker-positive and biomarker-negative patients eligible for a clinical trial). Biomarkers are produced endogenously and can be highly variable, especially in cross-sectional studies with significantly low statistical power for comparisons [15]. A further crucial step will be to conduct analytical validation to confirm accuracy and reliability of the biomarkers, including results reproducibility, precision, detection, and quantitation limits. In AS and SpA in general, disease activity relies on patient-reported outcomes (such as BASDAI and ASDAS): its correlation with biomarkers makes their investigations even more challenging. Another major challenge in translating microRNAs in reliable biomarkers is to validate the findings in large and well-characterized patient cohorts and datasets. The inadequacy of validation cohorts limits the evaluation of any identified biomarkers. Several works have reported that the validation of microRNA biomarkers and their translation to clinics is largely unsuccessful [76]. This low rate of success could be explained by different aspects: (i) differences in the study methodology, including pre-and post-analytical variables; (ii) the lack of standardized methods for normalization; (iii) the incapability to discriminate among closely related microRNAs [77].

In conclusion, further functional studies are needed in clarifying the role of specific microRNAs on gene function and their impact on specific cell subsets and this may be obtained also by investigating samples different from peripheral blood, as from entheseal or synovial tissue and fluid.
